# Reservoir computing for network intrusion classification

**DOI:** 10.1371/journal.pone.0355211

**Published:** 2026-08-14

**Authors:** Khorshed Alam, Mahbubul Haq Bhuiyan, Mohammad Ashraful Hoque, Mohammad Shohel Rana, Dewan Md. Farid

**Affiliations:** 1 Department of Computer Science and Engineering, Southeast University, Dhaka, Bangladesh; 2 Department of Computer Science and Engineering, United International University, Dhaka, Bangladesh; Maulana Abul Kalam Azad University of Technology West Bengal, INDIA

## Abstract

Network Intrusion Detection Systems (NIDS) play a critical role in securing IoT environments, where resource constraints demand lightweight yet effective solutions. While Convolutional Neural Network (CNN) and Long Short-Term Memory (LSTM) networks are widely adopted as benchmarks for intrusion detection, their high computational demands pose challenges for real-time deployment on IoT devices. Deep learning has emerged as a powerful approach for intrusion detection, and to explore lightweight alternatives, we investigate reservoir computing models, namely Echo State Networks (ESNs) and Liquid State Machines (LSMs). In the proposed framework, ESN and LSM act as temporal feature-learning and attack-classification engines, leveraging fixed recurrent reservoirs to efficiently capture network traffic dynamics while requiring minimal training overhead. We propose custom ESN- and LSM-based architectures that offer significantly lower computational complexity than conventional deep learning models. Our models demonstrate performance comparable to CNN and LSTM-based approaches while achieving substantial reductions in resource usage, making them suitable for real-time intrusion detection in IoT networks. We utilize the latest NF-ToN-IoT dataset from the University of Queensland, comprising 1,379,274 network flows spanning diverse attack categories. Furthermore, despite their potential advantages, ESN and LSM remain relatively underexplored in NIDS applications. The results highlight the viability of reservoir computing as an efficient, scalable, and responsive alternative for lightweight intrusion detection in IoT environments.

## 1. Introduction

The rise of IoT devices is bringing convenience and connectivity, however it is also creating a larger attack surface for cyber threats or intrusion. These devices often operate in resource-constrained environments which makes it difficult to deploy traditional security measures. Network Intrusion Detection Systems (NIDS) are crucial, however many of the state-of-the-art models are computationally heavy. Models like Deep Neural Networks (DNNs), Convolutional Neural Networks (CNNs) and Long Short-Term Memory networks (LSTMs) have shown strong performance in identifying malicious activity [1–3], but their demand for processing power and memory limits real-time application, especially at the edge.

Lightweight models are essential for IoT-based NIDS due to the limited computational resources, memory, and energy available on most IoT devices. In contrast to the traditional IT infrastructure, IoT nodes and, in particular, those deployed at the network edge require significant memory resources that are sometimes insufficient to run deep and complex models such as CNNs or LSTMs on a real-time basis. The heavy models may bring latencies, consume power and fail to scale well on distributed systems. The previous research [4,5] has repeatedly demonstrated that the computational requirements of deep learning solutions, such as CNNs and LSTMs, pose a challenge to jotting them onto an IoT setting despite the fact that these solutions are significantly more accurate in intrusion detection than the alternative. Scientists have also emphasized the issue of the complexity of the model versus its practicality in edge devices. Numerous works highlight the necessity of high-speed and energy-efficient models that should work in real-time and fundamentally do not need to be dependent on cloud infrastructure. Alternative architectures with a trade-off between performance and efficiency have also been of increasing interest but the majority of work remains to exploit the traditional deep learning models, and not to replace them with essentially simpler models such as reservoir computing. This is a blank area in the literature where further work can be undertaken to further explore inherently efficient model that fit the peculiar constraints of IoT.

The motivation of this study stems from the growing need for intrusion detection models that can operate efficiently in resource-constrained IoT environments without sacrificing detection performance. While existing studies predominantly focus on computationally intensive deep learning architectures such as CNNs and LSTMs, comparatively little attention has been given to reservoir-computing approaches for IoT-based NIDS. This research addresses that gap by investigating customized ESN and LSM-based intrusion detection frameworks that exploit fixed dynamic reservoirs and train only lightweight readout layers. Unlike conventional deep learning models that require optimization of all network parameters, the proposed approach aims to preserve competitive intrusion detection performance while significantly reducing computational complexity, memory requirements, and inference overhead. The objective of this work is therefore to evaluate whether reservoir-computing architectures can serve as practical and efficient alternatives to widely adopted deep learning benchmarks for real-time IoT intrusion detection. Our contributions are stated below:

- We propose custom lightweight architectures based on ESN, and LSM tailored for IoT-based Network Intrusion Detection Systems.
- We demonstrate that the proposed ESN and LSM models achieve performance comparable to widely used CNN and LSTM benchmarks in intrusion detection tasks.
- We achieve significant reduction in computational overhead, enabling practical, real-time deployment on resource-constrained IoT devices.
- We highlight the effectiveness of reservoir computing models for intrusion detection, an area that remains underexplored in existing literature.
- We conduct an ablation study on the ESN and LSM models to analyze the impact of key hyperparameters and provide guidance for future research and model optimization.

This paper is organized as follows. The Introduction presents the background and motivation for the study. The Literature Review summarizes related works and highlights the research gap. The Methodology section describes the proposed approach and experimental setup. The Results and Discussion section reports the findings and interprets their significance. The Limitations section outlines the constraints of the current work. Finally, the Conclusion summarizes the main contributions and suggests directions for future research.

## 2. Literature review

Recent innovations have driven users toward cloud environments, increasing the need for efficient NID systems. The detection of novel or unknown attacks is a problem with traditional methods because they result in high false positive rates and it is also difficult to overcome overfitting. Samriya et al. [6] employs a hybrid approach combining Ant Colony Optimization (ACO) for dimensionality reduction and DNN for classification, using preprocessed KDD Cup 99 and NSL-KDD datasets. Energy-efficient techniques, including min-max normalization and the 1-N encoding are part of 1-N encoding and DVFS. As experimental findings reveal, the ACO-DNN model is better in terms of accuracy and resource optimization compared to PCA-NB models. Nevertheless, the solution can be limited in scalability and may possibly be dependent on characteristics of a certain dataset, which does not necessarily apply to all datasets. Sun and Wang [7] proposed an image-based preprocessing method for network intrusion traffic data and utilized a modified LeNet model (ICNN-ID) for intrusion detection using NSL-KDD and CICIoV2024 datasets. The model achieved 89.97% and 99.996% accuracy respectively, outperforming 1D CNN and DCNN-BiLSTM models. Alrayes et al. [8] introduced IDS on the DNN model that reached a high value of validation accuracy (94.38%), precision, recall, and F1 score between normal and attack classes. With the model, the generalization and real-time threat identification properties are well exhibited. The study [9] propose an Improved Deep Neural Network (IDNN) optimized using the Coyote Optimization Algorithm (COA-GS) for intrusion detection in wireless sensor networks, using KDDCup 99 and WSN-DS datasets. The COA-GS-IDNN is better than the classical ML models having an accuracy of 95% of accuracy, 96% of recall and 98% of ROC AUC of detection and the time and delay of detection are low. Authors from [10] provided a multistage AI-based system with deep neural networks and transfer learning that enables robust intrusion detection, which can deal with zero-day attacks as well as adversarial attacks. The model attains the average accuracy of 98.5% on benchmark datasets indicating its wide-range adaptability against familiar and new threats. Constraints can be that it is computationally complex and only works in real-time.

A Hybrid AutoencoderResNet-LSTM model that makes use of the CNN, GRU, and LSTM architectures is proposed in this study [11] that aims to enhance a network in the context of detecting intrusions by downsizing data and based on the identified important parameters. Evaluated on NSL-KDD, UNSW-NB15, and CICIDS-2018 datasets, the model achieved high accuracy (up to 96.7%), outperforming existing methods. Author [12] suggested a deep learning-based intrusion detecting system based on the chaotic optimization strategy and Dugat-LSTM to recognize the attacks. Using TON-IOT and NSL-KDD datasets, the model achieves high accuracy (98.76% and 99.65% respectively) through advanced preprocessing, feature selection, and balancing techniques. While results show strong performance, the model’s complexity may limit its real-time applicability in low-resource environments. The work [13] introduces a new kind of anomaly detection system based on federated learning and blockchains to provide secure and resource-efficient IoT setting. Based on commitment coefficients and discrepancy between models, it can choose its best bet, namely, resilient devices to a model poisoning attack, to be part of FL process. Running on N-BaIoT dataset, the system is superior to FedProx and FedAvg both in terms of anomaly detection accuracy and robustness. Musthafa et al. [14] present an efficient ensemble method of IDS on IoT networks that could be deployed in the edge with a dual-stage compression strategy (pruning and quantization) on stacked LSTM with ANOVA feature selection. The system has been tested under the Kafka-based testbed, imitating real-life IoT settings, showing an accuracy rate of 97.3 percent with the efficiency being maintained with Raspberry Pi devices. Although it is successful, it is difficult to control high CPU utilization and scale to each type of edge hardware. The research work [15] introduces a lightweight intrusion detection system in IoT environments, comparing the deep learning architecture (e.g., MobileNetV3, EfficientNetB0) in image-based data and ML architectures on tabular information with the CIC-DDoS2019 dataset. Although DL models have large AUC results, their inference performance makes them not dependent on real time installation compared to ML models such as Random Forest model and SVM which are lightweight and can use limited resources on IoT devices. Xu et al. [16] offers a two-shoes multimodal approach of intrusion detection consisting of traffic feature graphs and network feature sets, with a CNN-based G-Model and a transformer-based S-Model. On CICIDS2017 and CICIDS2018 dataset, it reached 93.40 and 98.50 accuracy, which beat current solutions to few-shot tasks. Nonetheless, multimodal fusion complexity and problem of scalability to different network configurations are possible impediments. The paper [20] proposes one hybrid WOA-GWO method to execute IoT intrusion detection as it applies the combination of Whale and Grey Wolf Optimization options to optimize feature selection and parameters. The model has higher accuracies and responsiveness over the conventional methodologies such as LSTM-RNN, SVM, particularly in the detection of multi-class attacks. It has a lightweight structure that fits IoT environments with limited resources, but it can still present issues to translate into a variety of different environments in a real-time manner. A hybrid IDS approach is introduced by [17] in this study comprising of Local Outlier Factor (LOF) to locate the outliers and Convolutional Neural Network (CNN) to classify outliers with 99.87 percent accuracy on the CSE-CIC-IDS2018 dataset. It improves the detectability of anomalies because of both local density analysis enabled by LOF and feature extraction that CNN is based on, but their real-time applicability and their ability to generalize to previously unseen types of attacks are still to be investigated. Alashjaee [18] proposes the hybrid deep learning model Attention-CNN-LSTM, which has been obtained after integrating CNN, LSTM, and the self-attention mechanism to effectively detect intrusion. When tested on NSL-KDD and Bot-IoT datasets, it demonstrated an accuracy of 94–97 percent and an enhanced MCC and F1-scores and can run inference in real-time with low latency. Though auspicious in high trafficked environments, additional testing is required in a variety of network conditions. Alex et al. [19] experiment on ML and DL procedures with representative models RF, SVM, XGBoost, and a CNN-LSTM hybrid model to improve Network Intrusion Detection Systems. XGBoost had the best score of 99.80% and it was indeed effective in enhancing intrusion detection. It, however, still needs to be evaluated further under real-time conditions in order to check whether it can feasibly be deployed in practice.

A novel DNN-based intrusion detection system (IDS) has been proposed by [21] to strengthen the security of in-vehicular networks, where probability-based feature vectors extracted from network packets are used to train the model. Unlike traditional artificial neural networks, the approach leverages unsupervised pre-training with deep belief networks (DBN), thereby enhancing detection accuracy. Experimental results demonstrate its ability to provide real-time responses against malicious attacks in controller area networks (CAN) with a significantly improved detection ratio. Awad et al. [22] proposed an improved Long Short-Term Memory (ILSTM) algorithm for intrusion detection, where a conventional LSTM is first trained to obtain initial weights and then optimized using a hybrid of chaotic butterfly optimization (CBOA) and particle swarm optimization (PSO) to enhance accuracy. Two publicly available datasets NSL-KDD and LITNET-20 were considered to assess the methodology in terms of both binary and multi-class intrusion type classification. Outcomes showed that ILSTM performed better than the original LSTM, and other related deep-learning solutions with a higher accuracy of 93.09 and 96.86 precision than LSTM with 82.74 and 76.49 precision, but as it related to statistical significance, ILSTM proved significant. The disadvantages are the excessive complexity of computations since the hybrid algorithm optimization is involved, and the test is performed on only a few datasets, thus the need to conduct a follow-up test on the extended number of datasets to identify the possibility of having a broader application of the solution. In the paper [23], a hybrid intrusion detection model-IDS based on Fast R-CNN is proposed along with the dimensionality-reduction techniques of principal component analysis (PCA) and singular value decomposition (SVD). The system performs an assessment on the NIDS V.10 2017 dataset where the system had an accuracy rate of 99.5% which is better than the present machine-learning and deep-learning methods. The main weakness of this research is that it relies on one set of data to be tested and thus requires further testing in order to generalize. Yılmaz [24] proposed a novel deep-learning-based hybrid IDS that integrates three pre-trained models with Particle Swarm Optimization (PSO) for hyperparameter tuning to improve accuracy and efficiency. The methodology follows six stages, from data collection to evaluation, and is tested on KDDCUP’99, NSL-KDD, and UNSW-NB15 datasets. Results show accuracies of 93.55%, 90.42%, and 82.44%, respectively, outperforming existing approaches, though its effectiveness beyond benchmark datasets remains to be validated. Sun et al. [25] presented an integrated intrusion detection model for cluster-based wireless sensor networks, combining anomaly and misuse detection to improve detection rates and reduce false alarms. Anomaly detection is performed using a hierarchical Adaboost algorithm across sensor, cluster-head, and sink nodes, while misuse detection at the sink node leverages Back Propagation optimized with Cultural and Artificial Fish Swarm Algorithms. Simulation results demonstrate that the proposed model achieves strong intrusion detection performance, though validation in real-world deployments is needed. The investigation conducted by Alshahrani et al. [26] analyze adversarial machine learning in the framework of intrusion detection and tests the years of intrusion attacks maliciously produced by means of a GAN against the Decision Tree and the Logistic Regression. Each experiment uses the CICIDS2017 dataset and employs comparisons between the accuracy of models both before and after the attack. The findings show that the evasion attacks significantly decreased the accuracy of the detection, with the Decision Tree model being more vulnerable and poisoning attacks significantly distorted Logistic Regression training, yet the study has limitations, as it fails to cover a wider range of models and a wider range of classifiers, thus further validation has to be made in such contexts. Table 1 shows that most existing IoT intrusion detection approaches rely on computationally intensive deep learning architectures, hybrid optimization frameworks, or multimodal feature extraction mechanisms. Although these methods often achieve high detection accuracy, their complexity limits deployment in resource-constrained IoT environments. In contrast, reservoir computing approaches such as ESNs and LSMs remain largely unexplored for NIDS despite their inherent computational efficiency. This gap motivates the present study, which investigates lightweight ESN-DNN and LSM-DNN architectures for accurate and efficient intrusion detection in IoT networks.

**Table 1:** Summary of related work on IoT intrusion detection systems.

Ref.	Method	Dataset	Performance	Limitation
[6]	ACO-DNN	KDD Cup 99, NSL-KDD	Improved accuracy	Scalability and dataset dependency concerns
[7]	ICNN-ID	NSL-KDD, CICIoV2024	89.97%–99.99% Acc.	Computationally intensive image-based processing
[8]	DNN-based IDS	Public IDS dataset	94.38% Acc.	High computational overhead
[9]	COA-GS-IDNN	KDD Cup 99, WSN-DS	95% Acc., 96% Recall	Optimization increases complexity
[10]	Transfer Learning + DNN	Benchmark datasets	98.5% Acc.	High computational cost
[11]	Autoencoder-ResNet-LSTM	NSL-KDD, UNSW-NB15, CICIDS2018	96.7% Acc.	Complex hybrid architecture
[12]	Dugat-LSTM	TON-IoT, NSL-KDD	98.76%–99.65% Acc.	Limited suitability for low-resource devices
[13]	FL + Blockchain	N-BaIoT	Improved robustness	Deployment complexity
[14]	Compressed Stacked LSTM	IoT testbed	97.3% Acc.	High CPU utilization on edge devices
[15]	MobileNetV3, EfficientNetB0	CIC-DDoS2019	High AUC	Deep models remain computationally expensive
[16]	CNN + Transformer	CICIDS2017, CICIDS2018	93.4%–98.5% Acc.	Multimodal fusion complexity
[17]	LOF + CNN	CSE-CIC-IDS2018	99.87% Acc.	Generalization to unseen attacks unclear
[18]	Attention-CNN-LSTM	NSL-KDD, Bot-IoT	94%–97% Acc.	Requires further real-world validation
[19]	RF, SVM, XGBoost, CNN-LSTM	Public IDS datasets	99.8% Acc.	Real-time deployment not evaluated
**This Work**	**ESN-DNN, LSM-DNN**	**NF-ToN-IoT**	**94.75% Acc ESN; 93.63% Acc LSM with 77.68% fewer params than LSTM and 3.40% fewer than CNN**	**N/A**

## 3. Methodology

In this section, we discussed regarding data preparation, preprocessing and model development. Our approach is based on the principle of reservoir computing using Echo State Network and Liquid State Machine. We customized these architectures based on our project requirement. Let us break it down.

### 3.1 Data collection

In this research, we used the publicly accessible csv files in ToN-IoT dataset and extracted NetFlow data to create an IoT network data using NetFlow named NF-ToN-IoT, maintained by University of Queensland, Australia [27]. This dataset contains a total of 1,379,274 flow records whereby an enormous percentage of 80.4 percent (1, 108, 995 flows) of the records are characterized as attack traffic whereas the rest 19.6 percent (270,279 flows) are characterized as benign. The variety of threats that belong to attack classes include Injection (468,539 flows), DDoS (326,345), Password attacks (156,299), XSS (99,944), and the rest Scanning, Backdoor, DoS, MITM, and Ransomware. Every class shows the specific methods of compromising the IoT systems, and together they form a fully detailed basis of intrusion detection assessment in researching network security.

### 3.2 Data preprocessing

Data preprocessing starts with loading data into DataFrame as a CSV file. To increase privacy and possible leakage of data, sensitive columns (source and destination IP addresses IPV4_SRC_ADDR, and IPV4_DST_ADDR) are eliminated. The cleaned dataset is then divided and partitioned into training and testing datasets based on 80–20 division with a definite random seed to enable reproducibility.This split is encapsulated in a reusable function named simple_split. After splitting, both the training and testing datasets are cleaned further by removing any rows that contain missing (NaN) values, which helps ensure the quality and integrity of the data for subsequent machine learning tasks. The cleaned subsets are then printed to verify the preprocessing steps.

**Algorithm 1. Data Preprocessing for NF-ToN-IoT Dataset**

1: **Input:** NF-ToN-IoT dataset CSV file

2: Load dataset from NF-ToN-IoT.csv

3: Remove columns: IPV4_SRC_ADDR, IPV4_DST_ADDR, and Attack

4: **function** SimpleSplit (data)

5:  Split data into train_data and test_data with 80% and 20% respectively using fixed random seed

6:  **return** train_data, test_data

7: **end function**

8: train_data, test_data ← SimpleSplit(data)

9: Remove rows with missing values from train_data

10: Print train_data

11: Remove rows with missing values from test_data

12: Print test_data

### 3.3 Model development

The proposed NIDS framework utilizes two reservoir-computing architectures, namely Echo State Networks (ESNs) and Liquid State Machines (LSMs), as lightweight alternatives to conventional deep learning models. In both approaches, the reservoir serves as a temporal feature extraction mechanism that transforms sequential network traffic data into high-dimensional dynamic state representations, enabling the capture of complex temporal dependencies associated with normal and malicious activities. These reservoir states are subsequently processed by a trainable deep neural network (DNN) readout layer that performs the final intrusion classification. The ESN-based framework emphasizes efficient temporal memory through a fixed recurrent reservoir with controlled spectral dynamics, whereas the LSM-based framework introduces biologically inspired liquid-state dynamics that enhance the representation of transient and evolving network behaviors. Together, these architectures aim to achieve competitive intrusion detection performance while significantly reducing computational complexity, training overhead, and resource requirements for deployment in resource-constrained IoT environments.

#### 3.3.1 Echo State Network.

**Fig 1:**
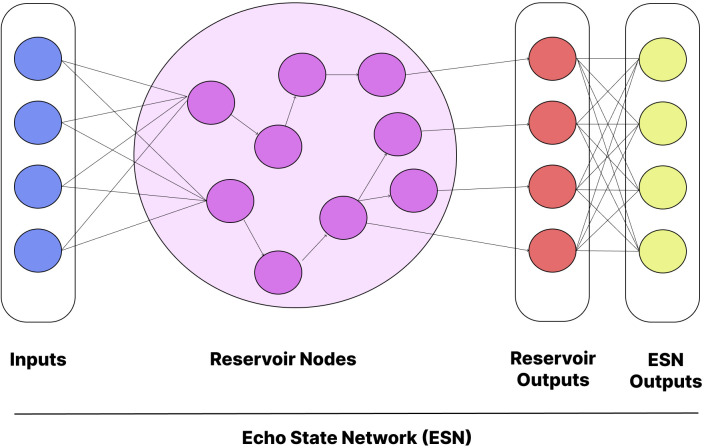
Echo State Network.

An Echo State Network (ESN) is a type of recurrent neural network where only the output weights are trained shown in fig 1. The reservoir, a large, fixed, and sparsely connected recurrent layer transforms the input into a dynamic, high-dimensional space. In this approach, a deep neural network (DNN) is used on top of the ESN reservoir to classify time series data. Let the input data at time step *t* be denoted as $u(t)\in {\mathbb{R}}^{d_{\text{in}}}$. The ESN consists of a reservoir of *N* neurons, with an internal state vector $x(t)\in {\mathbb{R}}^{N}$. The input weight matrix $W_{\text{in}}\in {\mathbb{R}}^{N\times d_{\text{in}}}$ and the recurrent reservoir weight matrix $W\in {\mathbb{R}}^{N\times N}$ are randomly initialized. Sparsity is enforced on *W*, and it is scaled to have a desired spectral radius ρ by:


$$\begin{array}{c} W\leftarrow \rho \cdot \frac{W}{\lambda_{\max}},\;\text{where }\lambda_{\max}=\max_{i}|\lambda_{i}|\text{ and }\lambda_{i}\text{ are eigenvalues of }W. \end{array}$$

**Algorithm 2. ESN Training and Prediction**

1: **Class** ManualESN(input_dim, reservoir_size, ρ, *sparsity*, α)

2: Initialize $W_{in}$, sparse *W*, scale *W* by ρ, bias, and zero state

3: **function** Update(*input*)

4:   $a\leftarrow W_{in}\cdot input+W\cdot state+bias$

5:   state←(1−α)·state+α·tanh(a)

6:   **return** *state*

7: **end function**

8: **function** FitTransform(*X, washout*)

9:   Reset *state*

10:   **for all** x∈X **do**

11:    state← Update(*x*)

12:    Store *state*

13:   **end for**

14:   **return** states[*washout*:]

15: **end function**

16: **function** Transform(*X*, *washout*)

17:   **for all** x∈X **do**

18:    state← Update(*x*)

19:    Store *state*

20:   **end for**

21:   **return** states[*washout*:]

22: **end function**

23: **Class** ESN_v2(reservoir_size, ρ, α)

24: **function** Train(*X, y, washout*)

25:   Create ManualESN

26:   H← FitTransform(*X*, *washout*)

27:   y←y[washout:]

28:   Define DNN with layers: [Dense-ReLU, Dropout, Dense-ReLU, Dense-Softmax]

29:   Compile and train DNN on (*H*, *y*)

30: **end function**

31: **function** Predict(*X, washout*)

32:   H← Transform(*X*, *washout*)

33:   **return** DNN.predict(*H*)

34: **end function**

The bias vector $b\in {\mathbb{R}}^{N}$ is also randomly initialized. The reservoir state is updated at each time step using a leaky integration mechanism with leakage rate α∈(0,1]:


$$\begin{array}{c} x(t)=(1-\alpha )\cdot x(t-1)+\alpha \cdot \tanh(W_{\text{in}}u(t)+Wx(t-1)+b). \end{array}$$

To prepare the input for the DNN, a sequence of input vectors {u(1),u(2),...,u(T)} is processed, and the corresponding reservoir states are collected into a matrix $X\in {\mathbb{R}}^{(T-w)\times N}$, after discarding the first *w* steps (washout period) to eliminate transient dynamics:


$$\begin{array}{c} X=[x(w+1),x(w+2),...,x(T){]}^{⊤}. \end{array}$$

The matrix *X* is then used as input to a deep neural network classifier. The DNN consists of multiple dense layers. The typical structure includes a first hidden layer with 64 ReLU units, followed by dropout, another dense layer with 32 ReLU units, and a final output layer with softmax activation. The output $\hat{y}$ represents the predicted class probabilities:


$$\begin{array}{c} h_{1}=\text{ReLU}(W_{1}X^{⊤}+b_{1}),\;h_{1}\leftarrow \text{Dropout}(h_{1},p=0.15), \end{array}$$

$$\begin{array}{c} h_{2}=\text{ReLU}(W_{2}h_{1}+b_{2}),\;\hat{y}=\text{Softmax}(W_{3}h_{2}+b_{3}). \end{array}$$

The DNN is trained using the sparse categorical cross-entropy loss function:


$$\begin{array}{c} ℒ=-\sum_{i=1}^{C}y_{i}\log({\hat{y}}_{i}), \end{array}$$

where $y_{i}$ is the true label and *C* is the number of classes. After training, predictions are made by transforming new input sequences using the same ESN and feeding the resulting state matrix to the trained DNN.

The ESN together with DNN have proven to be an efficient model to use in capturing the temporal dynamics in network traffic to be used in intrusion detection. In this method, the ESN is a dynamic reservoir that maps input data sequentially in a nonlinear and high dimensional space to enable temporal patterns, as well as anomalies to be more conveniently separated. The ESN requires less computation to train since the weights of the reservoir itself are not trainable but because the readout (in this case, a DNN) is fully trainable, the ESN still retains a memory of previous input. The DNN classifier also achieves a better performance due to the hierarchical nature of feature representations in the reservoir states which is ideal when it comes to multi-class as well as categorizing normal traffic into various network attacks. This architecture is based upon temporal dynamics and deep feature learning to enhance the matches of NIDS detector accuracy.

#### 3.3.2 Liquid State Network.

We proposed a reservoir computing based model inspired by Liquid State Machines, where a leaky recurrent reservoir projects input sequences into a high-dimensional state, followed by a deep neural network readout trained for classification. It is a type of recurrent neural network where a randomly connected, fixed-weight recurrent layer (the reservoir) transforms the input sequence into a dynamic high-dimensional space. This reservoir acts as a nonlinear temporal kernel, and the output of the reservoir can be used for various tasks, such as classification. In this implementation, the LSM output is passed to a DNN classifier for supervised learning. Let the input sequence be X={u(1),u(2),...,u(T)}, where each $u(t)\in {\mathbb{R}}^{d_{\text{in}}}$ is an input vector at time *t*. The reservoir consists of *N* neurons. Its internal state is denoted by $x(t)\in {\mathbb{R}}^{N}$. The reservoir is defined by:

- Input weight matrix: $W_{\text{in}}\in {\mathbb{R}}^{N\times d_{\text{in}}}$
- Recurrent weight matrix: $W\in {\mathbb{R}}^{N\times N}$ (sparse)
- Bias vector: $b\in {\mathbb{R}}^{N}$
- Leak rate: α∈(0,1]

The recurrent weights *W* are initialized randomly and sparsified according to a sparsity level s∈(0,1). The matrix is then scaled to have a spectral radius ρ=0.95:


$$\begin{array}{c} W\leftarrow \rho \cdot \frac{W}{\lambda_{\max}},\;\text{where }\lambda_{\max}=\max_{i}|\lambda_{i}|,\text{ the largest absolute eigenvalue of }W. \end{array}$$

The reservoir state is updated at each time step using a leaky integration rule with small stochastic perturbations to simulate liquid-like dynamics:


$$\begin{array}{c} x(t)=(1-\alpha )\cdot x(t-1)+\alpha \cdot \tanh(W_{\text{in}}u(t)+Wx(t-1)+b+\eta (t)), \end{array}$$

where $\eta (t)\sim 𝒩(0,\sigma^{2})$ is a small Gaussian noise vector added to introduce variability in the reservoir. The updated state is optionally clipped to the range [−1,1] to maintain stability and prevent saturation.

**Algorithm 3. LSM-Inspired Reservoir Training and Prediction**

1: **procedure** InitializeModel (*input dim, reservoir size, sparsity, α*)

2:   Set $state\leftarrow 0\in {\mathbb{R}}^{reservoir\_size}$

3:   $W_{in}\leftarrow \text{Uniform}(-1,1{)}^{reservoir\_size\times input\_dim}$

4:   $W\leftarrow \text{Uniform}(-1,1{)}^{reservoir\_size\times reservoir\_size}$

5:   Set W[i,j]←0 with probability 1−sparsity

6:   $W\leftarrow 0.95\cdot \frac{W}{\max(|\lambda |)}$ where λ are eigenvalues of *W*

7:   $bias\leftarrow \text{Uniform}(-0.5,0.5{)}^{reservoir\_size}$

8: **end procedure**

9: **procedure** UpdateState(*x*)

10:   $pre\leftarrow W_{in}\cdot x+W\cdot state+bias+\text{GaussianNoise}()$

11:   state←(1−α)·state+α·tanh(pre)

12:   state←clip(state,−1,1)

13:   **return** *state*

14: **end procedure**

15: **procedure** GenerateReservoirOutput(*X, reset*)

16:   **if** *reset* **then**

17:    state←0

18:   **end if**

19:   output←[]

20:   **for** each *x* in *X* **do**

21:    *output*.*append*(UpdateState(*x*))

22:   **end for**

23:   **return** *output*

24: **end procedure**

25: **procedure** Train(*X, y, epochs, batch_size*)

26:   R←GenerateReservoirOutput(X,reset=True)

27:   Initialize DNN:

28:    Dense(64, relu) → Dropout(0.15) → Dense(32, relu) → Dense(num_classes, softmax)

29:   Compile with RMSprop and sparse categorical crossentropy

30:   Fit DNN on *R* and *y* for given epochs and batch_size

31: **end procedure**

32: **procedure** Predict(*X*)

33:   R←GenerateReservoirOutput(X,reset=False)

34:   **return** DNN.predict(*R*)

35: **end procedure**

This process is applied for each input vector in the sequence, producing a sequence of reservoir states. These states are collected into a matrix:


$$\begin{array}{c} R=[\begin{array}{c} x(1{)}^{⊤}\\ x(2{)}^{⊤}\\ \vdots \\ x(T{)}^{⊤} \end{array}]\in {\mathbb{R}}^{T\times N}. \end{array}$$

The matrix *R* is then used as input to a deep neural network (DNN) for classification. The DNN typically includes the following layers:


$$\begin{array}{cc} \text{Layer 1:} & \;h_{1}=\text{ReLU}(W_{1}R^{⊤}+b_{1})\\ \text{Dropout:} & \;h_{1}\leftarrow \text{Dropout}(h_{1},p=0.15)\\ \text{Layer 2:} & \;h_{2}=\text{ReLU}(W_{2}h_{1}+b_{2})\\ \text{Output:} & \;\hat{y}=\text{Softmax}(W_{3}h_{2}+b_{3}) \end{array}$$

The model is trained using the sparse categorical cross-entropy loss:


$$\begin{array}{c} ℒ=-\sum_{i=1}^{C}y_{i}\log({\hat{y}}_{i}), \end{array}$$

where *C* is the number of output classes, *y* is the true class label (as a one-hot vector or sparse index), and $\hat{y}$ is the predicted class probability vector from the DNN.

At inference time shown in algorithm 3, the same reservoir transformation is applied to new inputs using the current reservoir state, and predictions are generated by feeding the resulting *R* into the trained DNN model.

**Algorithm 4. Measure Inference Time and Predict Labels**

1: **procedure** MeasureInferenceTime(*model, X*)

2:   start←current_time()

3:   preds←model.predict(X)

4:   end←current_time()

5:   total_time←end−start

6:   time_per_sample←total_time/len(X)

7:   Print “Inference time: total_time s total, time_per_sample s/sample”

8:   **return** *preds*

9: **end procedure**

10: $X_{\text{test}}\leftarrow \text{DropLabelColumn}(test\_data)$

11: $predictions\leftarrow \text{MeasureInferenceTime}(lsm\_model,X_{\text{test}})$

12: Print “Total trainable parameters in LSM: ” lsm_model.count_params()

Our LSM-inspired reservoir aligns with the theoretical principles of biological Liquid State Machines, in which recurrently connected neuronal microcircuits act as a high-dimensional temporal kernel. The sparse and random recurrent connectivity, coupled with leaky integration and small stochastic fluctuations, provides rich, nonlinear dynamics that allow the system to encode complex temporal patterns over multiple timescales. These dynamics reflect the computational behavior of cortical microcircuits, where transient internal states carry memory traces of recent inputs, enabling a separation of temporal information without requiring synaptic plasticity in the reservoir. By leveraging a deep neural network readout, the model extracts discriminative features from these high-dimensional states, implementing a biologically inspired, theoretically grounded framework for processing sequential and spatiotemporal data.

## 4. Results and discussion

### 4.1 Performance analysis

**Fig 2:**
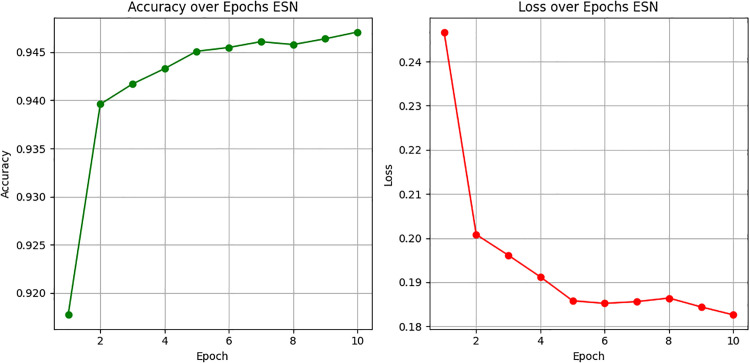
Accuracy and loss ESN.

We have performed ablation study for both ESN and LSM. We have also compared our proposed approach with widely used architectures such as Long Short Term Memory (LSTM) and Convolutional Neural Network. Our goal is to reach competitive performance like benchmark LSTM and CNN with less resources. Table 2 and Fig 2 show the measures of performance of ESN model in training and test data set. The model has been performing in a consistent way on both sets, and its accuracy on the training and test sets is quite high, around 94.77% and 94.75 percent respectively, which means that it is not overfitting. The precision, recall, and F1 score are more than 90 percent and are not too varied with training and test predictions, which implies the solidness of the model in both recognizing the attack and normal examples. Strong discriminatory power is also proven by the values of AUC-ROC, 0.9443 (train) and 0.9416 (test). To measure efficiency, the total inference time was also measured at 11.837345 seconds with an average time of only 0.000056 seconds per sample and the model can be used in a real-time or large-scale cases of intrusion detection.

**Table 2:** Performance metrics of the ESN model, Dataset = NF-ToN-IoT.

Metric	Train Data	Test Data
Accuracy	0.9477	0.9475
Precision	0.9095	0.9096
Recall	0.9054	0.9035
F1 Score	0.9075	0.9065
AUC-ROC	0.9443	0.9416

**Total Inference Time:** 11.837345 seconds.

**Average Time per Sample:** 0.000056 seconds.

**Total Trainable Parameters:** 18,210.

**Fig 3:**
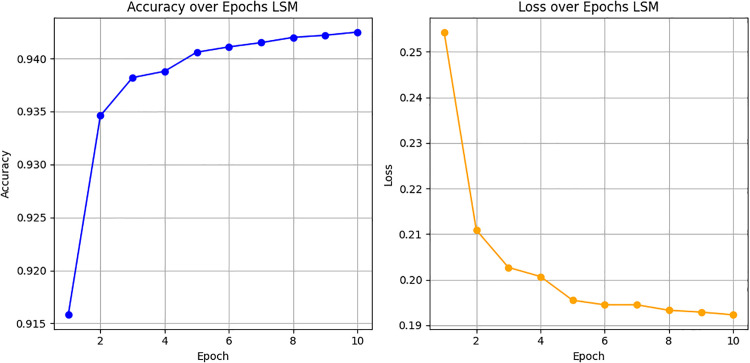
Accuracy and loss LSM.

The Table 3 and Fig 3 below give a summary of the performance measures of LSM model on training and test set. The model produces similar accuracy of 93.62 percent on training data and 93.63 percent on test data, which means that the generalization is reliable. All the precision, recall, and F1 scores are very favorable, they all reflect an acceptable 88.7% percent of accuracy in correctly detecting an intrusion and at the same time, not making many false alarms. The AUC-ROCs of more that 0.94 make the model state again that it has really high discriminative power of legitimate and malicious network traffic. Also, the running time to infer is about 12.45 seconds in total, and on average 0.000059 seconds of processing each sample, which points to the efficiency of the model that can be used in the real-time net intrusion detection system. The complexity of the model is medium having 18,210 trainable parameters and provides good balance between the performance and computational expense.

**Table 3:** Performance metrics of the LSM model, Dataset = NF-ToN-IoT.

Metric	Train Data	Test Data
Accuracy	0.9362	0.9363
Precision	0.8893	0.8896
Recall	0.8850	0.8836
F1 Score	0.8871	0.8866
AUC-ROC	0.9434	0.9423

**Total Inference Time:** 12.450968 seconds.

**Average Time per Sample:** 0.000059 seconds.

**Total Trainable Parameters:** 18,210.

### 4.2 Experimental setup

All experiments were conducted using the preprocessed NF-ToN-IoT dataset described in Section 3.2. The dataset was divided into training and testing subsets using an 80:20 split with a fixed random seed to ensure reproducibility. The input features were obtained by removing the target label column, while the Label column was used as the classification target.For the proposed ESN-based model, the reservoir size was set to 250, the spectral radius was set to 0.95, and the leakage rate α was set to 0.3. The ESN reservoir weights were randomly initialized and kept fixed during training, while only the DNN readout layer was optimized. The DNN readout consisted of a dense layer with 64 ReLU neurons, followed by a dropout layer with a dropout rate of 0.15, a second dense layer with 32 ReLU neurons, and a final softmax output layer for multi-class intrusion classification. The ESN-DNN model contained 18,210 trainable parameters.

For the LSM-based model, the input dimension was determined from the number of selected input features, and the reservoir size was set to 250. Similar to the ESN model, the LSM reservoir was used as a fixed recurrent temporal feature extractor, while the DNN readout layer was trained for classification. The LSM-DNN model used the same dense readout structure and contained 18,210 trainable parameters.

All neural models were trained for 10 epochs using the RMSprop optimizer and sparse categorical cross-entropy loss function. For comparative evaluation, LSTM and CNN models were also implemented as benchmark deep learning architectures. The LSTM model contained 81,570 trainable parameters, while the CNN model contained 18,850 trainable parameters. Model performance was evaluated using accuracy, precision, recall, F1-score, and AUC-ROC. Computational efficiency was further assessed using total inference time, average inference time per sample, and the number of trainable parameters.

### 4.3 Ablation study

**Fig 4:**
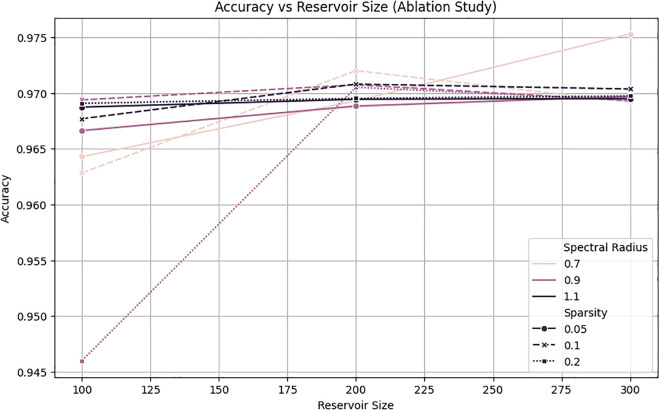
Accuracy Vs Reservoir size on ESN.

**Fig 5:**
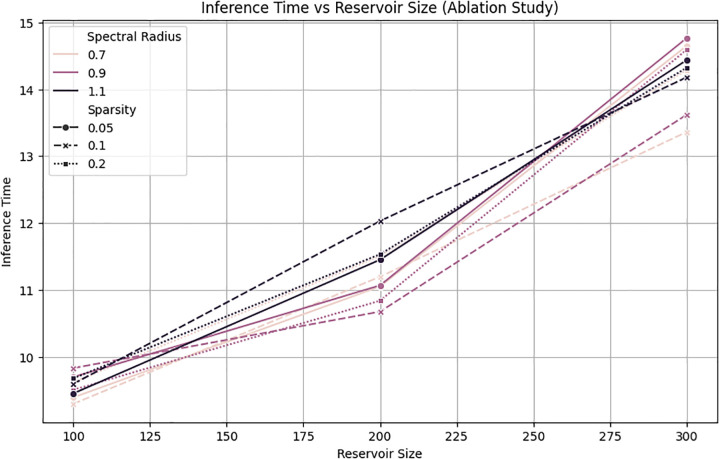
Inference time Vs Reservoir size on ESN.

We conducted an extensive ablation study to systematically evaluate the impact of three key hyperparameters of the ESN and LSM models: reservoir size, sparsity, and spectral radius (for ESN) or leakage rate α (for LSM). These parameters were initially chosen based on standard reservoir computing guidelines: reservoir sizes of 100, 200, and 300 to cover small to moderately large reservoirs; sparsity values between 0.05–0.2 to explore sparse versus dense connectivity; and spectral radii from 0.7–1.1 to ensure echo state property while allowing moderate memory retention. For LSM, α values of 0.2–0.5 were tested to study temporal memory decay effects. For the ESN model (Table 4 and Figs 4 and 5), we observed that predictive performance generally improves with larger reservoirs. The optimal configuration—reservoir size 300, sparsity 0.05, and spectral radius 0.7 achieved an accuracy of 0.9753 and F1 score of 0.9544. Denser reservoirs (lower sparsity) combined with moderate spectral radii (0.7–0.9) consistently outperformed other combinations, while extreme spectral radii (1.1 or very low values) slightly reduced performance. Increasing reservoir size naturally increased inference time (14.65 s for size 300) and the number of trainable parameters (21,410), highlighting a trade-off between computational cost and predictive accuracy.

**Table 4:** Ablation study results of ESN model, Dataset = NF-ToN-IoT.

Reservoir Size	Sparsity	Spectral Radius	Accuracy	F1 Score	Inference Time (s)	Trainable Params
100	0.05	0.7	0.9643	0.9311	9.39	8610
100	0.05	0.9	0.9666	0.9362	9.70	8610
100	0.05	1.1	0.9687	0.9405	9.45	8610
100	0.10	0.7	0.9628	0.9280	9.30	8610
100	0.10	0.9	0.9694	0.9417	9.83	8610
100	0.10	1.1	0.9677	0.9383	9.60	8610
100	0.20	0.7	0.9667	0.9362	9.62	8610
100	0.20	0.9	0.9460	0.8903	9.51	8610
100	0.20	1.1	0.9691	0.9411	9.68	8610
200	0.05	0.7	0.9693	0.9415	11.05	15010
200	0.05	0.9	0.9688	0.9408	11.07	15010
200	0.05	1.1	0.9694	0.9419	11.45	15010
200	0.10	0.7	0.9720	0.9473	11.20	15010
200	0.10	0.9	0.9707	0.9445	10.68	15010
200	0.10	1.1	0.9708	0.9448	12.03	15010
200	0.20	0.7	0.9687	0.9404	11.50	15010
200	0.20	0.9	0.9705	0.9441	10.84	15010
200	0.20	1.1	0.9695	0.9420	11.53	15010
300	0.05	0.7	**0.9753**	**0.9544**	14.65	21410
300	0.05	0.9	0.9697	0.9424	14.77	21410
300	0.05	1.1	0.9695	0.9420	14.44	21410
300	0.10	0.7	0.9692	0.9413	13.36	21410
300	0.10	0.9	0.9693	0.9418	13.62	21410
300	0.10	1.1	0.9704	0.9439	14.18	21410
300	0.20	0.7	0.9698	0.9435	14.29	21410
300	0.20	0.9	0.9693	0.9419	14.59	21410
300	0.20	1.1	0.9697	0.9424	14.32	21410

**Fig 6:**
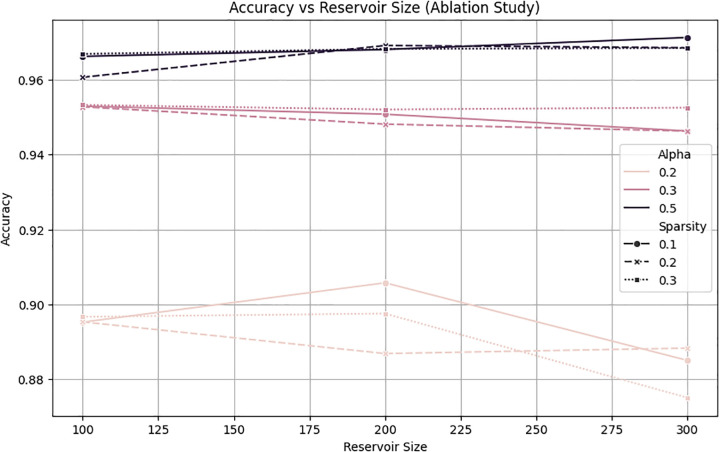
Accuracy Vs Reservoir size on LSM.

**Fig 7:**
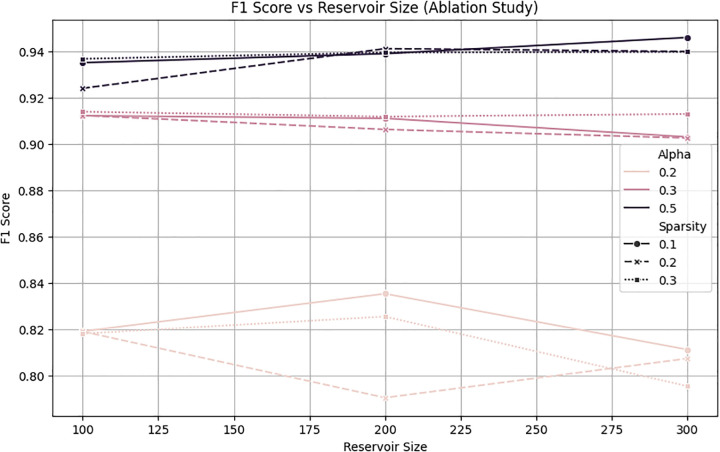
F1 score Vs Reservoir size on LSM.

For the LSM model (Table 5 and Figs 6 and 7), the effect of reservoir size, sparsity, and leakage rate α is more pronounced due to the spiking dynamics and temporal memory mechanisms. Performance improves with larger reservoirs: a reservoir size of 300 consistently outperformed smaller sizes (100 and 200), delivering up to 8–10% higher F1 scores. Leakage rate α strongly influences temporal memory: higher α (0.5) corresponds to faster decay and improved responsiveness to recent inputs, resulting in the best accuracy (0.9712) and F1 score (0.9460) for size 300 and sparsity 0.1. Lower α values (0.2–0.3) severely limited the model’s ability to capture sequential dependencies, yielding poor performance (accuracy 0.8851, F1 0.8112 for size 300). Sparsity also plays a critical role: moderate sparsity (0.1) balances the trade-off between connectivity and computational efficiency. Denser connectivity (0.1) ensures sufficient interactions among neurons to encode temporal features, while very high sparsity (0.3) can reduce effective network dynamics, slightly lowering accuracy (0.9525) and F1 (0.9130) even at the largest reservoir size. Inference time naturally increases with reservoir size and connectivity, reaching 14.08 s for the optimal configuration, but the parameter efficiency remains manageable (21,410 parameters).

**Table 5:** Full ablation study results of LSM model, Dataset = NF-ToN-IoT.

Reservoir Size	Sparsity	Alpha	Accuracy	F1 Score	Inference Time (s)	Params
100	0.1	0.2	0.8953	0.8191	10.07	8610
100	0.1	0.3	0.9529	0.9122	9.69	8610
100	0.1	0.5	0.9662	0.9351	9.60	8610
100	0.2	0.2	0.8953	0.8190	9.31	8610
100	0.2	0.3	0.9528	0.9124	9.40	8610
100	0.2	0.5	0.9606	0.9241	9.58	8610
100	0.3	0.2	0.8967	0.8181	9.68	8610
100	0.3	0.3	0.9532	0.9140	9.61	8610
100	0.3	0.5	0.9669	0.9368	9.61	8610
200	0.1	0.2	0.9058	0.8353	11.03	15010
200	0.1	0.3	0.9508	0.9111	10.90	15010
200	0.1	0.5	0.9680	0.9390	10.97	15010
200	0.2	0.2	0.8869	0.7904	10.60	15010
200	0.2	0.3	0.9481	0.9063	11.53	15010
200	0.2	0.5	0.9691	0.9412	10.88	15010
200	0.3	0.2	0.8976	0.8255	11.01	15010
200	0.3	0.3	0.9520	0.9118	11.07	15010
200	0.3	0.5	0.9682	0.9395	10.45	15010
300	0.1	0.2	0.8851	0.8112	12.96	21410
300	0.1	0.3	0.9463	0.9031	13.37	21410
300	0.1	0.5	**0.9712**	**0.9460**	14.08	21410
300	0.2	0.2	0.8883	0.8073	14.16	21410
300	0.2	0.3	0.9463	0.9026	13.45	21410
300	0.2	0.5	0.9685	0.9400	13.17	21410
300	0.3	0.2	0.8751	0.7954	12.18	21410
300	0.3	0.3	0.9525	0.9130	12.42	21410
300	0.3	0.5	0.9684	0.9398	13.43	21410

Taken together, the LSM ablation study quantitatively demonstrates that capturing temporal dependencies in sequential network intrusion data requires a careful balance of reservoir size, connectivity, and memory decay rate. Large reservoirs with moderate sparsity and higher leakage rates maximize both accuracy and F1 score, though with predictable increases in inference time. These results highlight the importance of architectural tuning in LSMs to optimize temporal feature representation while managing computational overhead.

Impact on Intrusion Detection Performance: The ablation results have direct implications for practical intrusion detection with our proposed ESN + DNN and LSM frameworks. In the ESN + DNN model, increasing reservoir size and reducing sparsity allow the network to capture richer temporal patterns in network traffic, which improves detection of attacks with prolonged temporal signatures such as DDoS or multi-stage scanning attacks. Similarly, tuning the spectral radius to moderate values (0.7–0.9) ensures sufficient memory of previous network states without introducing instability, enabling precise detection of bursts in traffic patterns associated with volumetric attacks.

For the LSM model, the leakage rate α critically affects the temporal decay of neuron activations, which directly influences the model’s sensitivity to short-lived versus long-lasting anomalies. Higher α (faster decay) allows the LSM to respond more promptly to sudden anomalies like XSS, whereas lower α can cause the network to overemphasize older activity, reducing detection accuracy for transient attacks. Moderate sparsity ensures that neurons interact enough to encode subtle temporal correlations across multiple attack vectors without excessive computational overhead. Collectively, these findings indicate that careful hyperparameter tuning not only maximizes overall accuracy and F1 score but also enhances the model’s ability to discriminate between attack types with distinct temporal characteristics.

#### 4.3.1 Prior study comparison.

The Table 6 comparison has been presented between four different neural networks or models which are widly used in prior work, such as LSTM, CNN, LSM, and ESN based on their classification performance and computation efficiency. CNN scored the best in the test accuracy (98.70%) and F1 score (97.67%), and closely behind with high recall (91.50%) and AUC-ROC (98.10%) is LSTM. Conversely, LSM and ESN did not show remarkably high accuracy (93.63% and 94.75% precisely), yet produced decent values of precision and F1. Being more precise, CNN and LSTM are nonetheless quite different in terms of model size and speed with CNN working faster and taking less place and LSTM being more complicated and slower. The LSM and ESN models are exceptional in regards to efficiency. Both forms have many fewer parameters to be trained (with numbers 18,210) than LSTM (81,570) and CNN (18,850), which is why they perform perfect enough to be deployed in resource-constrained environments. They are less than 13 seconds inference time on the entire test set, and sub-millisecond time per sample, though with simpler architectures than a typical transfer learning model, more than 93 percent accuracy. This trade-off in the expense of computation and predictive performance means that they have a place in real-time and edge situation where power, memory, and the time that anything has to run is often a high priority constraint.

**Table 6:** Comparison of model performance and computational efficiency, Dataset = NF-ToN-IoT.

Model	Test Acc.	Pre.	Recall	F1 Score	AUC-ROC	Train Acc.	Inference Time (s)	Trainable Params
LSTM [28–30]	0.9701	0.9794	0.9150	0.9435	0.9810	0.9704	9.974 / 0.000048	81,570
CNN [31,32]	0.9870	0.9831	0.9706	0.9767	0.9753	0.9872	7.427 / 0.000035	18,850
LSM	0.9363	0.8896	0.8836	0.8866	0.9423	0.9362	12.451 / 0.000059	18,210
ESN	0.9475	0.9096	0.9035	0.9065	0.9416	0.9477	11.837 / 0.000056	18,210

This trade-off consists in accuracy and resource efficiency. Although LSTM and CNN provide better prediction power, they are computationally and memory-intensive, especially, LSTM with more than 81K parameters and comparatively slower inferences. On the other hand, ESN and LSM are simpler and faster, and thus, more suitable applications, where light deployments are a priority, rather than peak accuracy. In this way, different applications should use specific types of models owing to the specific application context that requires high-stakes prediction tasks to use CNN or LSTM whereas embedded or real-time systems will be adversely affected by using ESN or LSM. All experiments were conducted using Python 3 on the Google Compute Engine backend, with system RAM usage averaging 4.3 GB of 12.7 GB available, and 21.4 GB of 107.7 GB disk space utilized.

## 5. Limitation

Despite their efficiency, ESN and LSM lack inherent mechanisms for online or continual learning, which may limit adaptability to evolving threats. However, if a DRL approach is integrated using ESN/LSM as RL agents, it could enable effective detection of zero-day attacks, a promising direction for future work.

## 6. Conclusion

The results of this study highlight the Echo State Network and Liquid State Machine as the most efficient and practical choices for real-world deployment, particularly in resource-constrained or real-time environments. While deep learning models like LSTM and CNN achieved slightly higher classification scores, they required significantly more trainable parameters and incurred longer training times. In contrast, ESN and LSM delivered competitive accuracy, precision, and F1 scores while maintaining extremely low computational overhead and faster inference times. This efficiency, paired with their robust performance, positions ESN and LSM as optimal solutions for edge computing and IoT-based intrusion detection, where energy and latency constraints are critical. Their balance of performance and efficiency makes them the true winners for scalable, real-time AI systems.

## 7. Resources

- Source Code: https://github.com/codewithkhurshed/ESNLSMIDS
- Dataset: https://staff.itee.uq.edu.au/marius/NIDS_datasets/
