# Supplementary material for: Reservoir computing for network intrusion classification
Source: PLoS One. 2026 Aug 14;21(8):e0355211. doi: 10.1371/journal.pone.0355211 (PMC13475880; doi:10.1371/journal.pone.0355211)
Supplement: Supplementary file 1 [file pone.0355211.s001.pdf]

Reservoir Computing for Network Intrusion Classification

General Feedback

The manuscript presents a timely and relevant study on using Echo State Networks (ESN) and Liquid State Machines (LSM) for lightweight IoT intrusion detection. The technical approach is sound, and the results are promising. However, the writing requires several minor grammatical and stylistic corrections to ensure clarity and professional presentation.

Specific Minor Corrections and Reviewer Comments

1. Title and Abstract

- **Abstract Grammar**: In the abstract, the phrase "resource constraints are demanding lightweight yet effective solutions" should be revised to "resource constraints **demand** lightweight yet effective solutions".
- **Abstract Clarity**: Change "their application in NIDS is remaining underexplored" to "their application in NIDS **remains** underexplored".

2. Introduction

- **Verb Tense**: Throughout the introduction, the authors use "is bringing," "is creating," and "is being". For a research article, it is standard to use the simple present or present perfect tense.
  - *Example*: Change "The rise of Internet of Things (IoT) devices is bringing convenience..." to "The rise of Internet of Things (IoT) devices **brings** convenience...".
- **Punctuation**: In Section 1, there is a missing comma before "however."
  - *Correction*: "...connectivity; however, it is also...".
- **Terminology**: Change "jotting them onto an IoT setting" to "**deploying** them in an IoT setting" for more formal academic tone.

3. Literature Review

- **Subject-Verb Agreement**: In paragraph 2, "Samriya et al. [6] employs..." should be "Samriya et al. [6] **employ**..." (as "et al." implies plural authors).
- **Consistency**: The text mentions "1-N encoding are part of 1-N encoding". This is redundant and should be rephrased for clarity.
- **Sentence Structure**: On page 10, the sentence "Alrayes et al. [8] introduced IDS on the DNN model that reached a high value..." should be revised to "**Alrayes et al. [8] introduced an IDS based on a DNN model that reached high values...**".

4. Methodology

- **Algorithm Description**: In Section 3.1, the text says "enormous percentage of 80.4 percent (1, 108, 995 flows) of the records are characterized as attack traffic". This can be simplified to: "**An 80.4% majority (1,108,995 flows) of the records represent attack traffic...**"
- **Equation Formatting**: Ensure all mathematical variables are consistently italicized in the text to match the equations .
- **Typo**: In Section 3.3.2, the word "intrufsions" is a typo and should be corrected to "**intrusions**".

5. Results and Discussion

- **Table References**: Ensure all tables are cited in the text as "Table 1" instead of "table 1".
- **Consistency in Decimals**: In Table 1, "0.9477" is used for accuracy, but the text later mentions "94.75 percent". It is better to stick to one format (either decimal or percentage) throughout the section.
- **Inference Time**: The text mentions "0.0" in the snippet provided for "Average Time per Sample" in Table 1. Ensure the full precision (e.g., 0.000056s) is displayed in the final table to provide meaningful data.

6. Figures and Captions

- **Completeness**: Figure 1 is cut off in the manuscript as "Fig 1. Echo". Ensure all figure captions are complete and descriptive.

Suggestion

The authors mention conducting an ablation study in the contributions. To strengthen the manuscript, ensure that the discussion clearly links how specific hyperparameters directly impact the detection of specific attack types like DDoS or XSS.
