# Supplementary material for: Reservoir computing for network intrusion classification
Source: PLoS One. 2026 Aug 14;21(8):e0355211. doi: 10.1371/journal.pone.0355211 (PMC13475880; doi:10.1371/journal.pone.0355211)
Supplement: Supplementary file 2 [file pone.0355211.s003.pdf]

1. If the authors have adequately addressed your comments raised in a previous round of review and you feel that this manuscript is now acceptable for publication, you may indicate that here to bypass the “Comments to the Author” section, enter your conflict of interest statement in the “Confidential to Editor” section, and submit your "Accept" recommendation.

Reviewer #1: All comments have been addressed

Author response: Thank you so much.

Reviewer #3: All comments have been addressed
Author response: Thank you so much.

2. Is the manuscript technically sound, and do the data support the conclusions?

The manuscript must describe a technically sound piece of scientific research with data that supports the conclusions. Experiments must have been conducted rigorously, with appropriate controls, replication, and sample sizes. The conclusions must be drawn appropriately based on the data presented.

Reviewer #1: Yes
Author response: Thank you so much.
Reviewer #3: Yes
Author response: Thank you so much.
3. Has the statistical analysis been performed appropriately and rigorously?

Reviewer #1: Yes
Author response: Thank you so much.
Reviewer #3: N/A
Author response: We have addressed your comments, thank you.
4. Have the authors made all data underlying the findings in their manuscript fully available?

The PLOS Data policy requires authors to make all data underlying the findings described in their manuscript fully available without restriction, with rare exception (please refer to the Data Availability Statement in the manuscript PDF file). The data should be provided as part of the manuscript or its supporting information, or deposited to a public repository. For example, in addition to summary statistics, the data points behind means, medians and variance measures should be available. If there are restrictions on publicly sharing data—e.g. participant privacy or use of data from a third party—those must be specified.

Reviewer #1: Yes
Author response: Thank you so much.
Reviewer #3: Yes
Author response: Thank you so much.
5. Is the manuscript presented in an intelligible fashion and written in standard English?

PLOS ONE does not copyedit accepted manuscripts, so the language in submitted articles must be clear, correct, and unambiguous. Any typographical or grammatical errors should be corrected at revision, so please note any specific errors here.

Reviewer #1: Yes
Author response: Dear R#1, Thank you so much.
Reviewer #3: Yes
Author response: Dear R#3, Thank you so much.
6. Review Comments to the Author

Please use the space provided to explain your answers to the questions above. You may also include additional comments for the author, including concerns about dual publication, research ethics, or publication ethics. (Please upload your review as an attachment if it exceeds 20,000 characters)

Reviewer #1: All comments have been addressed—every note reviewed, every fix applied. The final version is now clean, polished, and ready for the next step.
Author response: Dear R#1, Thank you so much.

Reviewer #3: This study proposed custom lightweight architectures based on Echo State Networks (ESN) and Liquid State Machines (LSM) tailored for IoT-based Network Intrusion Detection Systems (NIDS). The authors evaluated the proposed ESN- and LSM-based models using the NF-ToN-IoT dataset.
However, several important issues should be addressed before the manuscript can be considered for publication.

Abstract
• The abstract lacks sufficient clarity regarding the roles and objectives of ESN and LSM within the proposed framework. It is recommended to rewrite the abstract to establish a clearer and more coherent connection among the research background, identified research gap, methodology, and main contributions.

Author Response: Thank you for this valuable observation. To improve clarity, we revised the abstract to explicitly describe the roles of the Echo State Network (ESN) and Liquid State Machine (LSM) within the proposed NIDS framework. Specifically, we clarified that ESN and LSM serve as lightweight temporal feature-learning and attack-classification models that leverage fixed recurrent reservoirs to capture network traffic dynamics while maintaining low computational complexity and training overhead. This addition better communicates their objectives and contributions within the proposed intrusion detection framework.

Introduction

The motivation behind the study should be explicitly stated, along with a clear explanation of the novelty of the proposed approach compared with previous studies. In addition, the objectives and contributions of the paper should be clearly defined and aligned with the identified research gap and motivation. The manuscript currently claims an excessive number of contributions. It is recommended to focus on the main contributions to improve clarity, coherence, and readability.

Author Response: The Introduction has been revised to more explicitly state the study motivation, research gap, novelty, objectives, and main contributions, and the contribution list has been streamlined to focus on the core scientific contributions of the work.

Related Work

It is recommended to include a comparative summary table presenting the strengths and limitations of previous studies. Such a table would help readers better understand the research gaps addressed by the proposed work.

Author Response: Excellent review, a comparative summary table is included.

Methodology

The integration and utilization of ESN and LSM within the proposed contribution are currently unclear and require further explanation.

Author Response: Thank you for the valuable comment. The integration and utilization of ESN and LSM are described in Section 3.3 (Model Development), Section 3.3.1 (Echo State Network), and Section 3.3.2 (Liquid State Network), where the reservoir-computing framework, architectural design, mathematical formulations, training procedures, and algorithmic workflows are presented. In particular, Algorithms 2 and 3, together with the accompanying equations and architectural descriptions, explain how the reservoir states generated by ESN and LSM are utilized as inputs to the DNN readout layer for intrusion classification.

To further improve clarity, additional explanatory text has been added at the beginning of Section 3.3 to explicitly highlight the roles of ESN and LSM within the proposed NIDS framework.

Experimental Setup

• The experimental settings are not sufficiently described. The manuscript should include detailed parameter configurations and implementation settings for each employed algorithm or model to ensure reproducibility.

Author Response: Thank you for the suggestion. The manuscript already includes the principal model configurations and algorithmic settings within Sections 3.3.1 and 3.3.2,

including reservoir size, sparsity, spectral radius, leakage rate, network architecture, and training workflow. However, we agree that consolidating implementation details would improve reproducibility. Therefore, a dedicated Experimental Setup subsection has been added to summarize the training parameters, optimization settings, implementation environment, and configurations used in the experiments.

Results and Discussion

• The results section should be strengthened by providing deeper analysis and discussion of the obtained findings.

• A dedicated discussion section is recommended to better interpret the results, compare them with existing approaches, and highlight the practical significance of the study.

Statistical Analysis

• The significance and robustness of the reported results should be validated using appropriate statistical significance tests.

Author Response: Thank you for the comment. The manuscript already includes detailed interpretation of the obtained results in Sections 4.1 and 4.2. Beyond reporting performance metrics, the discussion examines model generalization through train-test comparisons, analyzes computational efficiency through inference time and parameter counts, and investigates the influence of key reservoir-computing hyperparameters through extensive ablation studies. Furthermore, the practical implications of reservoir size, sparsity, spectral radius, and leakage rate on intrusion detection performance are discussed in detail.
