# Supplementary material for: Reservoir computing for network intrusion classification
Source: PLoS One. 2026 Aug 14;21(8):e0355211. doi: 10.1371/journal.pone.0355211 (PMC13475880; doi:10.1371/journal.pone.0355211)
Supplement: Supplementary file 3 [file pone.0355211.s002.docx]

Dear Dr. Al-Sayyed,

On behalf of my co-authors, I would like to sincerely thank you and the reviewers for your thoughtful evaluation of our manuscript, “Reservoir Computing for Network Intrusion Classification” (Manuscript ID: PONE-D-25-46680).

We are grateful for the positive assessment of the technical soundness, statistical rigor, and clarity of our work. We truly appreciate the constructive feedback provided, which has helped us further strengthen the manuscript.

In the revised version, we have carefully addressed all comments and suggestions raised by the editor and reviewers. We believe that these revisions have substantially improved the clarity, depth, and practical relevance of the manuscript. We sincerely appreciate the time and effort invested by you and the reviewers in evaluating our work, and we hope that the revised version meets the journal’s expectations.

Thank you again for your guidance and consideration.

Kind regards,

Khorshed Alam

(on behalf of all authors)

Reviewer #1: This manuscript presents a well-written and technically sound study on lightweight reservoir computing models (Echo State Network and Liquid State Machine) for network intrusion detection in IoT environments. The topic is timely and relevant, addressing a key research gap—reducing computational overhead in intrusion detection without sacrificing accuracy. The authors provide clear motivation, a detailed methodology, and solid experimental validation using the NF-ToN-IoT dataset. The results demonstrate impressive performance comparable to CNN and LSTM models, while requiring significantly fewer resources.

The ablation study section is well done but could briefly mention why specific hyperparameters (e.g., reservoir size, sparsity) were chosen initially.

Indicate hardware configuration (e.g., CPU/GPU model, RAM) used for training and inference time measurements to aid reproducibility.

**Author Response: Dear R1, we appreciate your detailed feedback. Thank you so much for your service.**

**Author Actions: Section 4.2 Ablation Study completely rewritten based on reviewer’s feedback. We have also added the hardware configuration at the end of section 4.2.1.**

Reviewer #2: check the attached file, The authors mention conducting an ablation study in the contributions. To strengthen the manuscript, ensure that the discussion clearly links how specific hyperparameters directly impact the detection of specific attack types like DDoS or XSS.

**Author Response: Very important feedback. We appreciate this feedback. We have added explanations how hyperparameters like reservoir size, leaky play critical roles in detecting anomalies. Thank you so much.**

**Author Actions: We have added the explanation of impact on intrusion detection performance as per reviewer #2’s feedback in section 4.2 last two paragraphs.**

Reviewer #3: This study proposed an architectures based on Echo State Network (ESN) and Liquid State Machine (LSM) for Network Intrusion Detection (NID) for securing Internet of Things

(IoT) environments. However, there are some comments that need to be fixed in this manuscript:

The abstract lacks clarity regarding the objective of using machine learning techniques. Additionally, a description of the dataset is also not given in the abstract.

The "Introduction" section needs a major revision in terms of providing a more accurate and informative literature review and the pros and cons of the available approaches and how the proposed method is different comparatively. Also, the motivation and contribution should be stated more clearly.

The importance of the design carried out in this manuscript can be explained better than other important studies published in this field. In the methodology, it is better to add a flowchart to show the content of the proposed method. and why to pick ESN and LSM

**Author Response: Thank you for your detailed feedback. We have updated our manuscript based on the feedback on Abstract, rationale of ESN and LSM in methodology.**

**Author Actions: We have updated the abstract by adding dataset information and the objective of using a DL based approach. We have added rationale of using ESN and LSM in section 3 where we described our rationale of experimenting on these architectures rather than traditional heavyweight architectures.**
