# Supplementary material for: Reservoir computing for network intrusion classification
Source: PLoS One. 2026 Aug 14;21(8):e0355211. doi: 10.1371/journal.pone.0355211 (PMC13475880; doi:10.1371/journal.pone.0355211)
Supplement: Peer review history

# Peer review: Decision Letter 0

**DOI:** 10.1371/journal.pone.0355211.r001

**Authors:** Rizik M. H. Al-Sayyed

19 Feb 2026

PONE-D-25-46680

Reservoir Computing for Network Intrusion Classification

PLOS One

Dear Dr. Farid,

Thank you for submitting your manuscript to PLOS ONE. After careful consideration, we feel that it has merit but does not fully meet PLOS ONE’s publication criteria as it currently stands. Therefore, we invite you to submit a revised version of the manuscript that addresses the points raised during the review process.

Please submit your revised manuscript by Apr 05 2026 11:59PM. If you will need more time than this to complete your revisions, please reply to this message or contact the journal office at plosone@plos.org. When you're ready to submit your revision, log on to https://www.editorialmanager.com/pone/ and select the 'Submissions Needing Revision' folder to locate your manuscript file.

Please include the following items when submitting your revised manuscript:

- A letter that responds to each point raised by the academic editor and reviewer(s). You should upload this letter as a separate file labeled 'Response to Reviewers'.
- A marked-up copy of your manuscript that highlights changes made to the original version. You should upload this as a separate file labeled 'Revised Manuscript with Track Changes'.
- An unmarked version of your revised paper without tracked changes. You should upload this as a separate file labeled 'Manuscript'.

-->If you would like to make changes to your financial disclosure, please include your updated statement in your cover letter. Guidelines for resubmitting your figure files are available below the reviewer comments at the end of this letter.

If applicable, we recommend that you deposit your laboratory protocols in protocols.io to enhance the reproducibility of your results. Protocols.io assigns your protocol its own identifier (DOI) so that it can be cited independently in the future. For instructions see: https://journals.plos.org/plosone/s/submission-guidelines#loc-laboratory-protocols. Additionally, PLOS ONE offers an option for publishing peer-reviewed Lab Protocol articles, which describe protocols hosted on protocols.io. Read more information on sharing protocols at https://plos.org/protocols?utm_medium=editorial-email&utm_source=authorletters&utm_campaign=protocols.

We look forward to receiving your revised manuscript.

Kind regards,

Rizik M. H. Al-Sayyed

Academic Editor

PLOS One

Journal Requirements:

When submitting your revision, we need you to address these additional requirements.

1. Please ensure that your manuscript meets PLOS ONE's style requirements, including those for file naming. The PLOS ONE style templates can be found at

https://journals.plos.org/plosone/s/file?id=wjVg/PLOSOne_formatting_sample_main_body.pdf and

https://journals.plos.org/plosone/s/file?id=ba62/PLOSOne_formatting_sample_title_authors_affiliations.pdf

2. Please upload a copy of Supporting Information Figure/Table/etc. Supplementary information "Source Code and Datasets" which you refer to in your text on page 18.

3. If the reviewer comments include a recommendation to cite specific previously published works, please review and evaluate these publications to determine whether they are relevant and should be cited. There is no requirement to cite these works unless the editor has indicated otherwise.

4. Please review your reference list to ensure that it is complete and correct. If you have cited papers that have been retracted, please include the rationale for doing so in the manuscript text, or remove these references and replace them with relevant current references. Any changes to the reference list should be mentioned in the rebuttal letter that accompanies your revised manuscript. If you need to cite a retracted article, indicate the article’s retracted status in the References list and also include a citation and full reference for the retraction notice.

Additional Editor Comments:

The reviewer finds the work to be technically sound, with appropriate statistical analysis and sufficient data support for the conclusions. The manuscript is clearly written and meets the journal’s scientific standards.

However, some minor issues should be addressed before the manuscript can be accepted.

(1) The reviewer noted that while an ablation study is mentioned among the manuscript’s contributions, its practical implications are not sufficiently discussed. Specifically: (a) Clarify how individual hyperparameters influence performance, (b) Explicitly link these parameters to detection effectiveness for specific attack types (e.g., DDoS, XSS)

(2) The reviewers suggest improving: (a) The connection between experimental findings and real-world cyberattack detection, (b) The interpretability of model behavior, (c) The explanation of why certain configurations perform better

(3) Some minor refinements were suggested to: (a) Improve clarity of contributions, (b) Enhance explanation of results, (c) Strengthen the narrative around model robustness

[Note: HTML markup is below. Please do not edit.]

Reviewers' comments:

Reviewer's Responses to Questions

**Comments to the Author**

1. Is the manuscript technically sound, and do the data support the conclusions?

The manuscript must describe a technically sound piece of scientific research with data that supports the conclusions. Experiments must have been conducted rigorously, with appropriate controls, replication, and sample sizes. The conclusions must be drawn appropriately based on the data presented.

Reviewer #1: Yes

Reviewer #2: Yes

Reviewer #3: Yes

**********

2. Has the statistical analysis been performed appropriately and rigorously?

Reviewer #1: Yes

Reviewer #2: Yes

Reviewer #3: Yes

**********

3. Have the authors made all data underlying the findings in their manuscript fully available?

The PLOS Data policy requires authors to make all data underlying the findings described in their manuscript fully available without restriction, with rare exception (please refer to the Data Availability Statement in the manuscript PDF file). The data should be provided as part of the manuscript or its supporting information, or deposited to a public repository. For example, in addition to summary statistics, the data points behind means, medians and variance measures should be available. If there are restrictions on publicly sharing data—e.g. participant privacy or use of data from a third party—those must be specified.

Reviewer #1: Yes

Reviewer #2: Yes

Reviewer #3: Yes

**********

4. Is the manuscript presented in an intelligible fashion and written in standard English?

PLOS ONE does not copyedit accepted manuscripts, so the language in submitted articles must be clear, correct, and unambiguous. Any typographical or grammatical errors should be corrected at revision, so please note any specific errors here.

Reviewer #1: Yes

Reviewer #2: Yes

Reviewer #3: Yes

**********

5. Review Comments to the Author

Please use the space provided to explain your answers to the questions above. You may also include additional comments for the author, including concerns about dual publication, research ethics, or publication ethics. (Please upload your review as an attachment if it exceeds 20,000 characters)

Reviewer #1: Overall Evaluation:

This manuscript presents a well-written and technically sound study on lightweight reservoir computing models (Echo State Network and Liquid State Machine) for network intrusion detection in IoT environments. The topic is timely and relevant, addressing a key research gap—reducing computational overhead in intrusion detection without sacrificing accuracy. The authors provide clear motivation, a detailed methodology, and solid experimental validation using the NF-ToN-IoT dataset. The results demonstrate impressive performance comparable to CNN and LSTM models, while requiring significantly fewer resources.

The paper is suitable for publication after minor corrections to improve clarity, consistency, and presentation quality.

Strengths:

Focuses on resource-efficient intrusion detection, an important challenge in IoT cybersecurity.

Employs reservoir computing architectures (ESN, LSM) with detailed implementation and ablation studies.

Demonstrates strong experimental results (accuracy ~94–97%) with low inference latency.

Clear structure and logical flow from motivation to conclusion.

Publicly shared code and data ensure reproducibility.

Minor Revisions Required:

Language and Grammar

A few grammatical and stylistic issues exist (e.g., missing articles, inconsistent verb tenses, and long sentences).

A light proofreading or language polishing pass is recommended to enhance readability.

Figures and Tables

Some figures (e.g., Figures 2–7) should have larger axis labels and improved resolution for better readability.

Ensure consistent numbering and proper referencing of all figures and tables within the text.

Reference Consistency

Verify reference formatting according to PLOS ONE style—ensure consistency in capitalization, DOI format, and citation punctuation.

Include recent 2024–2025 references in relevant sections for completeness, especially in the Literature Review.

Methodological Clarity

The ablation study section is well done but could briefly mention why specific hyperparameters (e.g., reservoir size, sparsity) were chosen initially.

Indicate hardware configuration (e.g., CPU/GPU model, RAM) used for training and inference time measurements to aid reproducibility.

Presentation Improvements

The abstract and conclusion are strong, but could benefit from slightly shorter and more concise sentences to emphasize the main outcomes (accuracy, efficiency, applicability).

Consider adding a brief discussion paragraph comparing trade-offs between ESN/LSM and deep learning methods in practical IoT contexts.

Reviewer #2: check the attached file, The authors mention conducting an ablation study in the contributions. To strengthen the manuscript, ensure that the discussion clearly links how specific hyperparameters directly impact the detection of specific attack types like DDoS or XSS.

Reviewer #3: This study proposed an architectures based on Echo State Network (ESN) and Liquid State Machine (LSM) for Network Intrusion Detection (NID) for securing Internet of Things

(IoT) environments. However, there are some comments that need to be fixed in this manuscript:

The abstract lacks clarity regarding the objective of using machine learning techniques. Additionally, a description of the dataset is also not given in the abstract.

The "Introduction" section needs a major revision in terms of providing a more accurate and informative literature review and the pros and cons of the available approaches and how the proposed method is different comparatively. Also, the motivation and contribution should be stated more clearly.

The importance of the design carried out in this manuscript can be explained better than other important studies published in this field.

In the methodology, it is better to add a flowchart to show the content of the proposed method. and why to pick ESN and LSM

in the results section, it is better to compare the obtained results with state of the art

and to show the significance of the obtained results comparing to them

**********

6. PLOS authors have the option to publish the peer review history of their article (what does this mean?). If published, this will include your full peer review and any attached files.

If you choose “no”, your identity will remain anonymous but your review may still be made public.

**Do you want your identity to be public for this peer review?**  For information about this choice, including consent withdrawal, please see our Privacy Policy.

Reviewer #1: No

Reviewer #2: No

Reviewer #3: **Yes:**  Dheeb Albashish

**********

[NOTE: If reviewer comments were submitted as an attachment file, they will be attached to this email and accessible via the submission site. Please log into your account, locate the manuscript record, and check for the action link "View Attachments". If this link does not appear, there are no attachment files.]

To ensure your figures meet our technical requirements, please review our figure guidelines: https://journals.plos.org/plosone/s/figures

You may also use PLOS’s free figure tool, NAAS, to help you prepare publication quality figures: https://journals.plos.org/plosone/s/figures#loc-tools-for-figure-preparation.

NAAS will assess whether your figures meet our technical requirements by comparing each figure against our figure specifications.

Attachment Submitted filename: Reservoir Computing for Network Intrusion Classification.pdf

# Author comment: Author response to Decision Letter 1

**DOI:** 10.1371/journal.pone.0355211.r002

11 Mar 2026

Response to Reviewers file is attached with this submission.

Attachment Submitted filename: Response to Reviewers-PONE-D-25-46680.docx

# Peer review: Decision Letter 1

**DOI:** 10.1371/journal.pone.0355211.r003

**Authors:** Sohail Saif

14 May 2026

PONE-D-25-46680R1

Reservoir Computing for Network Intrusion Classification

PLOS One

Dear Dr. Farid,

Thank you for submitting your manuscript to PLOS ONE. After careful consideration, we feel that it has merit but does not fully meet PLOS ONE’s publication criteria as it currently stands. Therefore, we invite you to submit a revised version of the manuscript that addresses the points raised during the review process.

Please submit your revised manuscript by Jun 28 2026 11:59PM. If you will need more time than this to complete your revisions, please reply to this message or contact the journal office at plosone@plos.org. When you're ready to submit your revision, log on to https://www.editorialmanager.com/pone/ and select the 'Submissions Needing Revision' folder to locate your manuscript file.

Please include the following items when submitting your revised manuscript:

- A letter that responds to each point raised by the academic editor and reviewer(s). You should upload this letter as a separate file labeled 'Response to Reviewers'.
- A marked-up copy of your manuscript that highlights changes made to the original version. You should upload this as a separate file labeled 'Revised Manuscript with Track Changes'.
- An unmarked version of your revised paper without tracked changes. You should upload this as a separate file labeled 'Manuscript'.

If you would like to make changes to your financial disclosure, please include your updated statement in your cover letter. Guidelines for resubmitting your figure files are available below the reviewer comments at the end of this letter.

If applicable, we recommend that you deposit your laboratory protocols in protocols.io to enhance the reproducibility of your results. Protocols.io assigns your protocol its own identifier (DOI) so that it can be cited independently in the future. For instructions see: https://journals.plos.org/plosone/s/submission-guidelines#loc-laboratory-protocols. Additionally, PLOS ONE offers an option for publishing peer-reviewed Lab Protocol articles, which describe protocols hosted on protocols.io. Read more information on sharing protocols at https://plos.org/protocols?utm_medium=editorial-email&utm_source=authorletters&utm_campaign=protocols.

As the corresponding author, your ORCID iD is verified in the submission system and will appear in the published article. PLOS supports the use of ORCID, and we encourage all coauthors to register for an ORCID iD and use it as well. Please encourage your coauthors to verify their ORCID iD within the submission system before final acceptance, as unverified ORCID iDs will not appear in the published article. *Only*  the individual author can complete the verification step; PLOS staff *cannot*  verify ORCID iDs on behalf of authors.

We look forward to receiving your revised manuscript.

Kind regards,

Sohail Saif, Ph.D

Academic Editor

PLOS One

Journal Requirements:

If the reviewer comments include a recommendation to cite specific previously published works, please review and evaluate these publications to determine whether they are relevant and should be cited. There is no requirement to cite these works unless the editor has indicated otherwise.

[Note: HTML markup is below. Please do not edit.]

Reviewers' comments:

Reviewer's Responses to Questions

**Comments to the Author**

1. If the authors have adequately addressed your comments raised in a previous round of review and you feel that this manuscript is now acceptable for publication, you may indicate that here to bypass the “Comments to the Author” section, enter your conflict of interest statement in the “Confidential to Editor” section, and submit your "Accept" recommendation.

Reviewer #1: All comments have been addressed

Reviewer #3: All comments have been addressed

**********

2. Is the manuscript technically sound, and do the data support the conclusions?

The manuscript must describe a technically sound piece of scientific research with data that supports the conclusions. Experiments must have been conducted rigorously, with appropriate controls, replication, and sample sizes. The conclusions must be drawn appropriately based on the data presented.

Reviewer #1: Yes

Reviewer #3: Yes

**********

3. Has the statistical analysis been performed appropriately and rigorously?

Reviewer #1: Yes

Reviewer #3: N/A

**********

4. Have the authors made all data underlying the findings in their manuscript fully available?

The PLOS Data policy requires authors to make all data underlying the findings described in their manuscript fully available without restriction, with rare exception (please refer to the Data Availability Statement in the manuscript PDF file). The data should be provided as part of the manuscript or its supporting information, or deposited to a public repository. For example, in addition to summary statistics, the data points behind means, medians and variance measures should be available. If there are restrictions on publicly sharing data—e.g. participant privacy or use of data from a third party—those must be specified.

Reviewer #1: Yes

Reviewer #3: Yes

**********

5. Is the manuscript presented in an intelligible fashion and written in standard English?

PLOS ONE does not copyedit accepted manuscripts, so the language in submitted articles must be clear, correct, and unambiguous. Any typographical or grammatical errors should be corrected at revision, so please note any specific errors here.

Reviewer #1: Yes

Reviewer #3: Yes

**********

6. Review Comments to the Author

Please use the space provided to explain your answers to the questions above. You may also include additional comments for the author, including concerns about dual publication, research ethics, or publication ethics. (Please upload your review as an attachment if it exceeds 20,000 characters)

Reviewer #1: All comments have been addressed—every note reviewed, every fix applied. The final version is now clean, polished, and ready for the next step.

Reviewer #3: This study proposed custom lightweight architectures based on Echo State Networks (ESN) and Liquid State Machines (LSM) tailored for IoT-based Network Intrusion Detection Systems (NIDS). The authors evaluated the proposed ESN- and LSM-based models using the NF-ToN-IoT dataset.

However, several important issues should be addressed before the manuscript can be considered for publication.

Abstract

• The abstract lacks sufficient clarity regarding the roles and objectives of ESN and LSM within the proposed framework.

• It is recommended to rewrite the abstract to establish a clearer and more coherent connection among the research background, identified research gap, methodology, and main contributions.

Writing Style

• The overall writing style requires significant improvement. The manuscript frequently employs first-person expressions such as “We have utilized,” which should be replaced with a more formal academic writing style.

Introduction

• The introduction section requires major revision. A more comprehensive and up-to-date literature review should be provided, highlighting both the advantages and limitations of existing approaches.

• The motivation behind the study should be explicitly stated, along with a clear explanation of the novelty of the proposed approach compared with previous studies.

• In addition, the objectives and contributions of the paper should be clearly defined and aligned with the identified research gap and motivation.

• The manuscript currently claims an excessive number of contributions. It is recommended to focus on the main contributions to improve clarity, coherence, and readability.

Related Work

• It is recommended to include a comparative summary table presenting the strengths and limitations of previous studies. Such a table would help readers better understand the research gaps addressed by the proposed work.

Methodology

• A detailed framework or flowchart should be added to clearly illustrate the overall architecture and workflow of the proposed system.

• The integration and utilization of ESN and LSM within the proposed contribution are currently unclear and require further explanation.

Experimental Setup

• The experimental settings are not sufficiently described. The manuscript should include detailed parameter configurations and implementation settings for each employed algorithm or model to ensure reproducibility.

Results and Discussion

• The results section should be strengthened by providing deeper analysis and discussion of the obtained findings.

• A dedicated discussion section is recommended to better interpret the results, compare them with existing approaches, and highlight the practical significance of the study.

Statistical Analysis

• The significance and robustness of the reported results should be validated using appropriate statistical significance tests.

**********

7. PLOS authors have the option to publish the peer review history of their article (what does this mean?). If published, this will include your full peer review and any attached files.

If you choose “no”, your identity will remain anonymous but your review may still be made public.

**Do you want your identity to be public for this peer review?**  For information about this choice, including consent withdrawal, please see our Privacy Policy.

Reviewer #1: No

Reviewer #3: **Yes:**  Dheeb Albashish

**********

[NOTE: If reviewer comments were submitted as an attachment file, they will be attached to this email and accessible via the submission site. Please log into your account, locate the manuscript record, and check for the action link "View Attachments". If this link does not appear, there are no attachment files.]

To ensure your figures meet our technical requirements, please review our figure guidelines: https://journals.plos.org/plosone/s/figures

You may also use PLOS’s free figure tool, NAAS, to help you prepare publication quality figures: https://journals.plos.org/plosone/s/figures#loc-tools-for-figure-preparation.

NAAS will assess whether your figures meet our technical requirements by comparing each figure against our figure specifications.

# Author comment: Author response to Decision Letter 2

**DOI:** 10.1371/journal.pone.0355211.r004

28 Jun 2026

Response to Reviewers file is attached.

Attachment Submitted filename: Response to Reviewers.pdf

# Peer review: Decision Letter 2

**DOI:** 10.1371/journal.pone.0355211.r005

**Authors:** Sohail Saif

19 Jul 2026

Reservoir Computing for Network Intrusion Classification

PONE-D-25-46680R2

Dear Dr. Farid,

We’re pleased to inform you that your manuscript has been judged scientifically suitable for publication and will be formally accepted for publication once it meets all outstanding technical requirements.

Within one week, you’ll receive an e-mail detailing the required amendments. When these have been addressed, you’ll receive a formal acceptance letter and your manuscript will be scheduled for publication.

An invoice will be generated when your article is formally accepted. Please note, if your institution has a publishing partnership with PLOS and your article meets the relevant criteria, all or part of your publication costs will be covered. Please make sure your user information is up-to-date by logging into Editorial Manager at Editorial Manager® and clicking the ‘Update My Information' link at the top of the page. For questions related to billing, please contact billing support.

If your institution or institutions have a press office, please notify them about your upcoming paper to help maximize its impact. If they’ll be preparing press materials, please inform our press team as soon as possible -- no later than 48 hours after receiving the formal acceptance. Your manuscript will remain under strict press embargo until 2 pm Eastern Time on the date of publication. For more information, please contact onepress@plos.org.

Kind regards,

Sohail Saif, Ph.D

Academic Editor

PLOS One

Additional Editor Comments (optional):

Reviewers' comments:

Reviewer's Responses to Questions

**Comments to the Author**

1. If the authors have adequately addressed your comments raised in a previous round of review and you feel that this manuscript is now acceptable for publication, you may indicate that here to bypass the “Comments to the Author” section, enter your conflict of interest statement in the “Confidential to Editor” section, and submit your "Accept" recommendation.

Reviewer #1: All comments have been addressed

**********

2. Is the manuscript technically sound, and do the data support the conclusions?

The manuscript must describe a technically sound piece of scientific research with data that supports the conclusions. Experiments must have been conducted rigorously, with appropriate controls, replication, and sample sizes. The conclusions must be drawn appropriately based on the data presented.

Reviewer #1: Yes

**********

3. Has the statistical analysis been performed appropriately and rigorously?

Reviewer #1: Yes

**********

4. Have the authors made all data underlying the findings in their manuscript fully available?

The PLOS Data policy requires authors to make all data underlying the findings described in their manuscript fully available without restriction, with rare exception (please refer to the Data Availability Statement in the manuscript PDF file). The data should be provided as part of the manuscript or its supporting information, or deposited to a public repository. For example, in addition to summary statistics, the data points behind means, medians and variance measures should be available. If there are restrictions on publicly sharing data—e.g. participant privacy or use of data from a third party—those must be specified.

Reviewer #1: Yes

**********

5. Is the manuscript presented in an intelligible fashion and written in standard English?

PLOS ONE does not copyedit accepted manuscripts, so the language in submitted articles must be clear, correct, and unambiguous. Any typographical or grammatical errors should be corrected at revision, so please note any specific errors here.

Reviewer #1: Yes

**********

6. Review Comments to the Author

Please use the space provided to explain your answers to the questions above. You may also include additional comments for the author, including concerns about dual publication, research ethics, or publication ethics. (Please upload your review as an attachment if it exceeds 20,000 characters)

Reviewer #1: accept all comments have been addressed accept all comments have been addressed accept all comments have been addressed

**********

7. PLOS authors have the option to publish the peer review history of their article (what does this mean?). If published, this will include your full peer review and any attached files.

If you choose “no”, your identity will remain anonymous but your review may still be made public.

**Do you want your identity to be public for this peer review?**  For information about this choice, including consent withdrawal, please see our Privacy Policy.

Reviewer #1: No

**********

# Editor report: Acceptance letter

**DOI:** 10.1371/journal.pone.0355211.r006

**Authors:** Sohail Saif

PONE-D-25-46680R2

PLOS One

Dear Dr. Farid,

I'm pleased to inform you that your manuscript has been deemed suitable for publication in PLOS One. Congratulations! Your manuscript is now being handed over to our production team.

At this stage, our production department will prepare your paper for publication. This includes ensuring the following:

* All references, tables, and figures are properly cited

* All relevant supporting information is included in the manuscript submission,

* There are no issues that prevent the paper from being properly typeset

You will receive further instructions from the production team, including instructions on how to review your proof when it is ready. Please keep in mind that we are working through a large volume of accepted articles, so please give us a few days to review your paper and let you know the next and final steps.

Lastly, if your institution or institutions have a press office, please let them know about your upcoming paper now to help maximize its impact. If they'll be preparing press materials, please inform our press team within the next 48 hours. Your manuscript will remain under strict press embargo until 2 pm Eastern Time on the date of publication. For more information, please contact onepress@plos.org.

You will receive an invoice from PLOS for your publication fee after your manuscript has reached the completed accept phase. If you receive an email requesting payment before acceptance or for any other service, this may be a phishing scheme. Learn how to identify phishing emails and protect your accounts at https://explore.plos.org/phishing.

If we can help with anything else, please email us at customercare@plos.org.

Thank you for submitting your work to PLOS ONE and supporting open access.

Kind regards,

PLOS ONE Editorial Office Staff

on behalf of

Dr. Sohail Saif

Academic Editor

PLOS One
